# Xiebai San Alleviates Allergic Pulmonary Inflammation by Modulating Arachidonic Acid Metabolism

**DOI:** 10.3390/ph19030440

**Published:** 2026-03-09

**Authors:** Xingnan Yue, Jiayin Han, Chen Pan, Yushi Zhang, Suyan Liu, Feng Gao, Yong Zhao, Jingwen Wu, Yuhao Wang, Xi Cheng, Aihua Liang

**Affiliations:** Key Laboratory of Beijing for Identification and Safety Evaluation of Chinese Medicine, Institute of Chinese Materia Medica, China Academy of Chinese Medical Sciences, Beijing 100700, China; yuexingnan1226@163.com (X.Y.); jyhan@icmm.ac.cn (J.H.); cpan@icmm.ac.cn (C.P.); yszhang@icmm.ac.cn (Y.Z.); syliu@icmm.ac.cn (S.L.); fgao@icmm.ac.cn (F.G.); yzhao@icmm.ac.cn (Y.Z.); nnniwu333@gmail.com (J.W.); wangyuhao1089@163.com (Y.W.); xicheng572@gmail.com (X.C.)

**Keywords:** Xiebai San, p38 MAPK pathway, arachidonic acid, acute allergic pulmonary inflammation, chronic allergic pulmonary inflammation

## Abstract

**Background:** Xiebai San (XBS), a classical Traditional Chinese Medicine formula comprising *Cortex mori*, *Lycii Radicis Cortex*, and *Glycyrrhizae Radix et Rhizoma*, has long been used for pulmonary inflammatory disorders. However, its underlying mechanisms remain un-clear. This study aimed to investigate the mechanisms by which XBS alleviates allergic pulmonary inflammation. **Methods:** Two murine models were established, consisting of a chronic ovalbumin (OVA)-induced model simulating adaptive immune responses and an acute compound 48/80-induced model triggering non-IgE-dependent mast cell activation. Pharmacodynamic indices including serum IgE, histamine, inflammatory cytokines, leukocyte profiles, and lung histopathology were evaluated. Network pharmacology was employed to predict core pathways. Arachidonic acid metabolites (AAMs) in lung tissues were quantified by targeted UPLC-MS/MS, and p38 MAPK signaling proteins were assessed by Western blot. **Results:** XBS significantly alleviated lung injury in both models. In the chronic OVA-induced model, XBS significantly reduced serum immunoglobulin E levels and inflammatory cell infiltration. In the acute model, XBS suppressed histamine release and mast cell-mediated inflammatory responses. Targeted metabolomics revealed differential regulatory mechanisms: XBS reduced lipoxygenase-derived metabolites, including leukotrienes and 12-hydroxyeicosatetraenoic acid in chronic inflammation, while suppressing cyclooxygenase-related prostaglandins in acute inflammation. Network pharmacology analysis identified arachidonic acid (AA) metabolism as a potential central pathway. The p38 mitogen-activated protein kinase pathway was partially involved. **Conclusions:** XBS effectively alleviates both chronic and acute allergic pulmonary inflammation through differential modulation of AA metabolism, providing mechanistic insights supporting its traditional use in allergic airway diseases.

## 1. Introduction

Allergic pulmonary inflammation is a respiratory disorder characterized by dysregulated immune responses, airway remodeling, and impaired lung function. It can manifest as acute bronchoconstriction or chronic, irreversible pathological changes such as goblet cell hyperplasia and subepithelial fibrosis [1]. This condition, particularly prevalent among children and young adults, imposes a substantial global health burden, affecting approximately 260 million people in 2021 and projected to reach 275 million by 2050 [2,3,4]. Current pharmacotherapies, including corticosteroids, antihistamines, and biologics targeting IgE or IL-5R, provide symptomatic relief but fail to address the underlying immune dysregulation, leading to incomplete responses, tachyphylaxis, systemic side effects, and poor long-term adherence [5,6].

Among the key molecular mechanisms underlying allergic pulmonary inflammation, arachidonic acid (AA) metabolism plays a central role in orchestrating allergic inflammation. Upon activation, cytosolic phospholipase A_2_ (cPLA_2_) hydrolyzes membrane phospholipids to release free AA, which is subsequently converted via cyclooxygenase (COX), lipoxygenase (LOX), and cytochrome P450 into a wide array of lipid mediators, including prostaglandins, thromboxanes, leukotrienes, and hydroxyeicosatetraenoic acids (HETEs) [7,8,9]. These lipid mediators regulate vascular permeability, immune-cell recruitment, mast cell activation, mucus hypersecretion, and airway remodeling, thereby critically contributing to the pathogenesis of allergic pulmonary inflammation. Immediate responses in allergic pulmonary inflammation, such as mast cell and basophil degranulation, rapidly release histamine, leukotrienes, and prostaglandins, causing bronchoconstriction and vascular leakage. In contrast, chronic inflammation is driven by persistent cytokine and lipid mediator release [10]. Dysregulated COX/LOX activity is strongly associated with both chronic IgE-mediated inflammation and acute mast cell-driven hypersensitivity responses. The mitogen-activated protein kinase (MAPK), particularly p38MAPK, directly regulates cPLA_2_ activation and thereby governs downstream AA metabolites [11,12]. Aberrant MAPK activation amplifies inflammatory mediator production and sustains airway inflammation.

Given the pivotal role of AA metabolism in allergic pulmonary inflammation, therapeutic agents capable of modulating this pathway may provide significant clinical benefits. Xiebai San (XBS), a classical prescription first recorded in the Song Dynasty text Xiao Er Yao Zheng Zhi Jue (c. 11th century) [13], comprises *Cortex mori* (the dried root bark of *Morus alba* L.), *Lycii Radicis Cortex* (the dried root bark of *Lycium barbarum* L.), and *Glycyrrhizae Radix et Rhizoma* (the dried root and rhizome of *Glycyrrhiza uralensis* Fisch.) Its phytochemical constituents include flavonoids (e.g., morusin, sanggenon) and organic acids with documented anti-inflammatory and immunomodulatory effects [14,15,16]. XBS has been shown to alleviate key pathological features such as leukocyte infiltration and goblet cell hyperplasia, improve lung function, and reduce circulating IL-6 and TNF-α levels. Importantly, it exhibits a favorable safety profile [17,18,19,20,21]. Given these broad anti-inflammatory effects, understanding the molecular pathways through which XBS modulates immune responses is essential for elucidating its therapeutic mechanism.

In the present study, we employ the ovalbumin (OVA) model to recapitulate chronic allergic inflammation with IgE-mediated mast cell activation and eosinophilia [22,23] and the compound 48/80 (C48/80) model to capture acute mast cell-driven inflammation with rapid mediator release and vascular leakage [24]. This study investigates the mechanisms through which XBS attenuates both chronic and acute allergic pulmonary inflammation.

## 2. Results

### 2.1. Chemical Characterization of XBS

**Figure 1:**
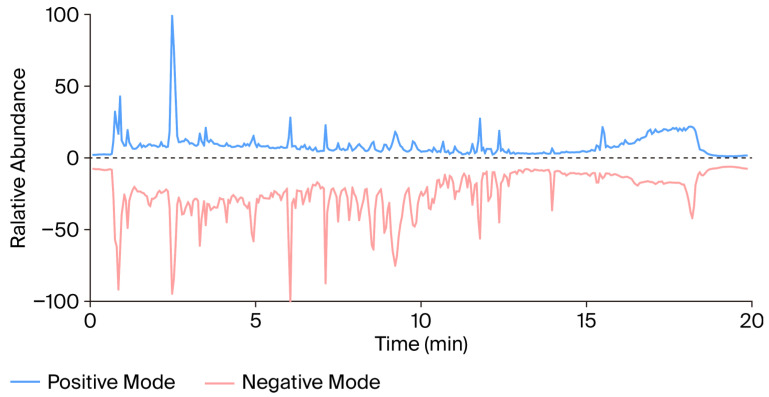
Chemical characterization of Xiebai San (XBS) by UHPLC-QTOF-MS.

To characterize the chemical composition of Xiebai San (XBS), a comprehensive analysis was performed using UHPLC-QTOF-MS. The representative base peak chromatograms (BPCs) in both positive and negative ion modes are presented in Figure 1. By comparing the retention times and high-resolution MS/MS fragmentation patterns with those of authentic standards, as well as consulting established databases and literature, five major compounds were identified or tentatively identified: glycyrrhizic acid, liquiritin, chlorogenic acid, kukoamine B, and mulberroside A (Table 1). These represent key bioactive compound classes from the constituent herbs.

Base peak chromatograms (BPCs) in positive (top) and negative (bottom) ion modes. Five major compounds identified by comparing retention times and MS/MS fragmentation patterns with authentic standards: glycyrrhizic acid, liquiritin, chlorogenic acid, kukoamine B, and mulberroside A (see Table 1 for detailed MS data).

### 2.2. XBS Protects Lung Tissues Against OVA-Induced Allergic Pulmonary Inflammation in Mice

**Figure 2:**
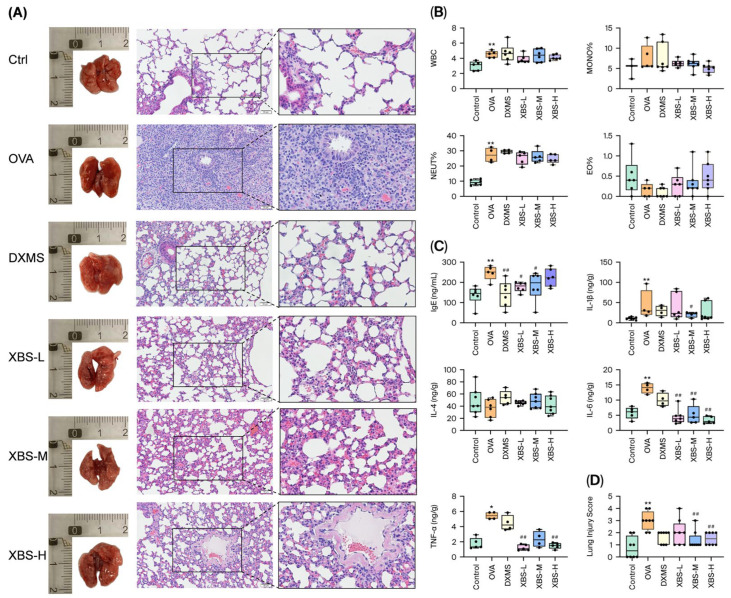
XBS alleviated OVA-induced allergic pulmonary inflammation in mice. (**A**) HE staining of lung tissue. (scale bar = 100 μm) (**B**) Total white blood cell (WBC) counts and WBC differential counts including neutrophils (NEUT%), monocytes (MONO%), and eosinophils (EO%). (**C**) Levels of inflammatory cytokines (TNF-α, IL-1β, IL-6) in lung tissue homogenates and serum IgE. (**D**) Lung injury scores based on the semi-quantitative grading system described in Table 2. Data are presented as mean ± SD (*n* = 6 mice per group). All parameters were assessed 24 h after the final OVA challenge (day 19). *n* = 6. Colored boxes represent different experimental groups, and each dot represents an individual mouse. Rectangles in panel (A) indicate regions shown at higher magnification.* *p* < 0.05 and ** *p* < 0.01 compared with the control group; # *p* < 0.05 and *## p* < 0.01 compared with the OVA group.

A chronic allergic airway inflammation model was established by intraperitoneal sensitization with OVA plus aluminum hydroxide followed by repeated intranasal OVA challenges. The protective effects of XBS against OVA-induced allergic pulmonary inflammation in mice were evaluated. Lung tissues and blood samples were collected 24 h after the final OVA challenge (day 19). Histopathological assessment (Figure 2A–D) revealed severe lung injury in the OVA group compared with the control, characterized by inflammatory cell infiltration, thickened septa, narrowed alveolar lumen, and focal eosinophilic infiltration. XBS treatment (XBS-L, XBS-M, XBS-H) markedly alleviated these lesions and normalized septal thickness to varying degrees. The lung injury scores (Figure 2) were markedly elevated in the OVA group relative to the control, whereas XBS- and dexamethasone-treated mice exhibited substantially lower scores.

At 24 h after the final OVA challenge (day 19), OVA exposure led to significant increases in total peripheral WBC counts, as well as NEUT%, MONO%, and EO%. Serum IgE levels were markedly elevated in OVA-challenged mice. XBS treatment effectively reversed these changes, reducing WBC, NEUT%, MONO% and EO%. The inflammatory response was further evidenced by elevated TNF-α, IL-1β and IL-6 levels in lung tissue and elevated serum IgE levels after OVA challenge (Figure 2C). XBS administration lowered serum IgE levels in all dose groups (Figure 2B). Histologically, XBS also alleviated lung tissue damage and significantly improved pathological scores based on a semi-quantitative grading system (Table 2), as shown in Figure 2D. Collectively, these findings demonstrate that XBS mitigates OVA-induced allergic pulmonary inflammation by alleviating histopathological damage, restoring hematological homeostasis, reducing serum IgE, and suppressing pro-inflammatory cytokine expression in lung tissue.

### 2.3. XBS Protects Lung Tissues Against C48/80-Induced Allergic Pulmonary Inflammation in Mice

**Figure 3:**
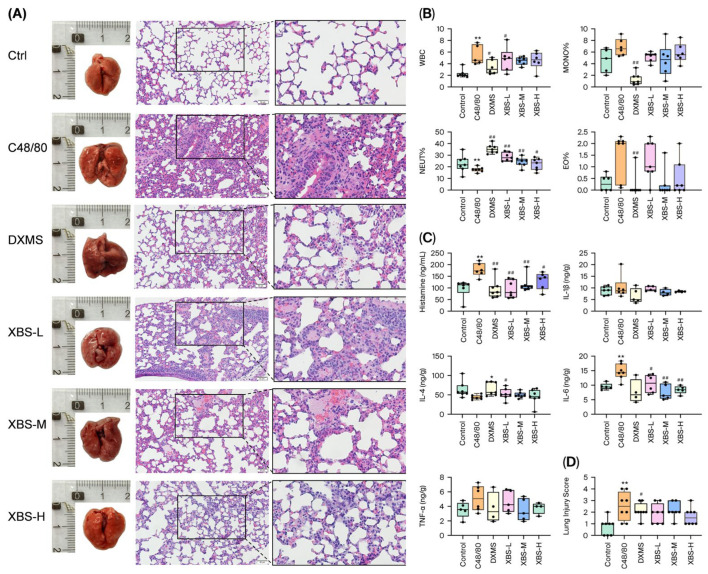
XBS alleviated C48/80-induced allergic pulmonary inflammation in mice. (**A**) HE staining of lung tissue. (scale bar = 100 μm) (**B**) Total white blood cell (WBC) counts and WBC differential counts including neutrophils (NEUT%), monocytes (MONO%), and eosinophils (EO%). (**C**) Levels of inflammatory cytokines (TNF-α, IL-1β, IL-6) in lung tissue homogenates and serum IgE. (**D**) Lung injury scores based on the semi-quantitative grading system described in Table 2. All parameters were assessed 30 min after C48/80 injection. *n* = 6. * *p* < 0.05 and ** *p* < 0.01 compared with the control group; # *p* < 0.05 and ## *p* < 0.01 compared with the C48/80 group.

An acute mast cell–mediated inflammation model was induced by tail vein injection of compound 48/80 following oral pretreatment with XBS or dexamethasone. The modulatory effects of XBS on C48/80-induced allergic responses in mice were evaluated. Lung tissues and blood samples were collected 30 min after C48/80 injection. As shown in Figure 3A–D, compared with the control group, C48/80-challenged mice exhibited marked lung injury, characterized by inflammatory cell infiltration and thickened alveolar septa. However, proteinaceous exudates were more prominent in the bronchioles of C48/80-challenged lungs, in contrast to eosinophilic exudates observed in the lungs of OVA-challenged mice. XBS administration alleviated this histopathological alteration. In all treatment groups, inflammatory cell infiltration was diminished, alveolar structure was restored, alveolar septa became thinner, and alveolar lumens enlarged, with more pronounced improvements observed in the medium and high-dose groups (XBS-M, XBS-H). Moreover, XBS improved lung tissue integrity and significantly ameliorated pathological scores following C48/80 challenge (Figure 3D).

At 30 min after C48/80 stimulation, total WBC counts, MONO%, and EO% were significantly increased, accompanied by changes in neutrophil proportions. XBS treatment counteracted these hematological alterations by reducing WBC, MONO%, and EO% levels (Figure 3B). The inflammatory cascade induced by C48/80 was further evidenced by elevated pulmonary cytokines (IL-6, TNF-α) and increased serum histamine. XBS effectively suppressed IL-6 and TNF-α expression in lung tissues at all tested doses (Figure 3C). Collectively, these results demonstrate that XBS mitigates C48/80-induced allergic responses by reducing histopathological damage, restoring hematological parameters, lowering serum histamine, and inhibiting pulmonary IL-6 and TNF-α expression in a dose-dependent fashion.

Together, these findings indicate that OVA-induced inflammation was primarily characterized by IgE elevation and leukotriene-mediated eosinophilic responses, whereas C48/80 induced rapid histamine release and prostaglandin-mediated acute inflammation. XBS showed protective effects in both models, but through distinct modulatory patterns.

### 2.4. Network Pharmacology Predicts Potential Targets and Pathways Associated with XBS in Allergic Pulmonary Inflammation

**Figure 4:**
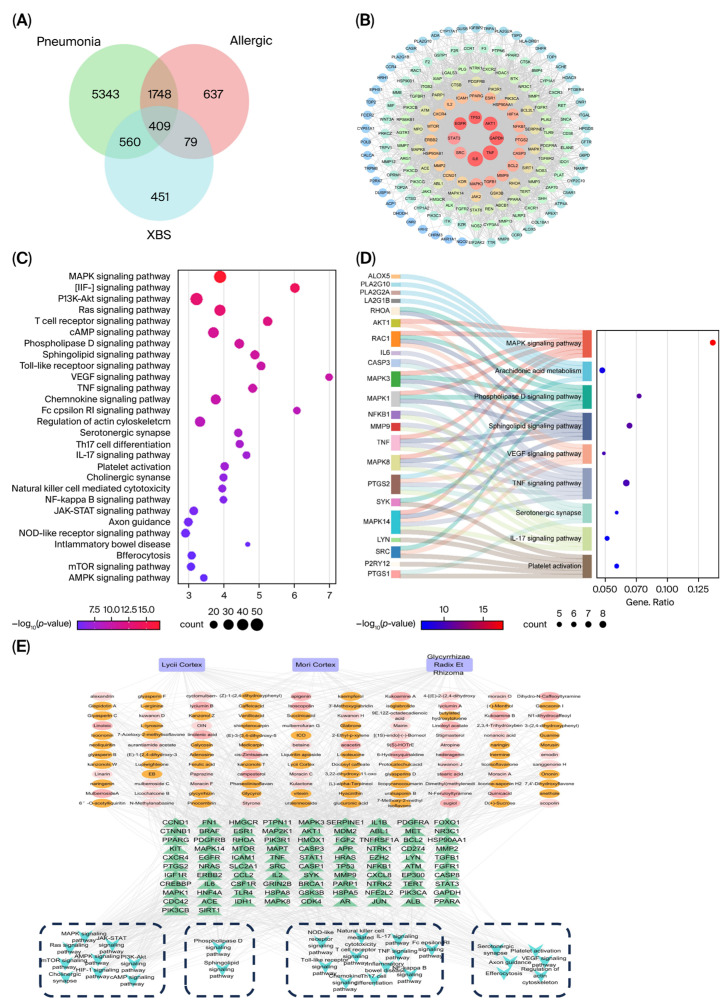
Network pharmacological analysis of XBS against allergic pulmonary inflammation. (**A**) Venn diagram of target overlap XBS, pneumonia, and allergy. (**B**) Protein–protein interaction (PPI) network of 171 overlapping targets (STRING confidence ≥0.4, visualized in Cytoscape). Node size indicates degree centrality. (**C**) Bar chart of top 20 enriched KEGG pathways of XBS. (**D**) Sankey diagram illustrating gene-pathway relationships. Left: core target genes; middle: enriched KEGG pathways; right: enrichment statistics. Ribbon width represents gene count per pathway. (**E**) The drugs-components-targets-Kegg Pathway network; purple nodes represent drugs, orange and pink nodes represent components, green nodes represent targets, and blue nodes represent Kegg Pathway. Edge thickness represents binding/interaction strength.

Through screening based on oral bioavailability (OB) ≥ 30% and drug-likeness (DL) ≥ 0.18, a total of 110 active ingredients of XBS were identified, including 33 from Sangbaipi, 14 from Digupi, and 63 from Gancao. A total of 895 potential targets of Sangbaipi, Digupi, and Gancao were predicted using the SwissTargetPrediction platform. A total of 1238 allergic pulmonary inflammation-related targets were collected from OMIM and the GeneCards database. Intersection with XBS predicted targets yielded 171 overlapping targets. The STRING database was used to obtain the topology of the 171 overlapping targets. A protein–protein interaction (PPI) network was constructed in Cytoscape and analyzed using the “Network Analyzer (version 2.8)” tool. Thirty-two core targets, including IL6, TNF, GAPDH, AKT1, TP53, EGFR, STAT3, SRC, and MAPK3, were identified. The “TCM-Active Ingredient-Target” network of XBS was constructed (Figure 4E). Network topology analysis identified the top core ingredients, including quercetin, kaempferol, glepidotin A, cyclomulberrochromene, sexangularetin, glyasperin B, kanzonolsW, and Jaranol. It should be noted that network pharmacology screening based on OB and DL criteria may exclude compounds with lower predicted bioavailability but high actual abundance. LC/MS analysis identified glycyrrhizic acid, liquiritin, chlorogenic acid, kukoamine B, and mulberroside A as major constituents in XBS (Figure 4). These compounds, particularly glycyrrhizic acid and liquiritin, are present at high concentrations and may contribute to the therapeutic effects through intestinal metabolism or direct local actions. Therefore, network pharmacology prediction and LC/MS-based chemical profiling provide complementary insights into the bioactive constituents of XBS.

GO enrichment analysis of the 32 core targets revealed 398 biological processes (BPs), 104 molecular functions (MFs), and 50 cellular components (CCs). BPs were mainly enriched in positive regulation of miRNA transcription, negative regulation of gene expression, and positive regulation of nitric oxide biosynthesis. CCs were mainly enriched in glutamatergic synapse, plasma membrane, nucleoplasm, and neuronal cell body. MFs were primarily associated with enzyme binding, protein kinase binding, protein kinase activity, and ubiquitin protein ligase binding. KEGG pathway enrichment analysis identified 149 pathways, and the top 20 pathways were ranked by *p*-value and presented (Figure 4C,D). The enrichment analysis indicated that XBS exerts anti-allergic pulmonary inflammation effects primarily by modulating multiple signaling pathways, including the MAPK, PI3K-Akt, IL-17, phospholipase D, sphingolipid, and platelet activation pathways. Notably, key enzymes directly involved in AA metabolism were identified among the core targets: ALOX5 (5-LOX), which catalyzes AA conversion to leukotrienes; PLA2G2A and PLA2G10, secretory phospholipases A_2_ responsible for AA release from membrane phospholipids; and PTGS1 (COX-1), enriched in both the serotonergic synapse and platelet activation pathways. Additionally, MAPK14 (p38 MAPK), a critical regulator of COX-2 expression, was enriched across multiple pathways along with MAPK1 (ERK2) and MAPK8 (JNK). At the upstream level, the phospholipase D signaling pathway, containing targets such as RHOA, RAC1, SRC, SYK, and LYN, may facilitate AA release through its metabolic product phosphatidic acid (PA), which is sequentially converted to diacylglycerol and subsequently liberates AA. The sphingolipid signaling pathway can further enhance PLA_2_ activity through sphingosine-1-phosphate signaling. At the level of AA metabolic conversion, the MAPK cascade (MAPK14, MAPK1, MAPK8) regulates both the expression and activity of downstream metabolic enzymes, while inflammation-related targets (TNF, IL6) in the TNF and IL-17 signaling pathways transcriptionally upregulate COX-2 and 5-LOX expression. This integrated bioinformatics analysis strongly suggested that XBS may exert anti-allergic effects by comprehensively modulating AA metabolism and its derived eicosanoids. To directly validate this prediction, targeted metabolomics analysis of AA and its metabolites was subsequently performed.

### 2.5. Modulation of Arachidonic Acid Metabolites by XBS in OVA-Induced Allergic Pulmonary Inflammation

**Figure 5:**
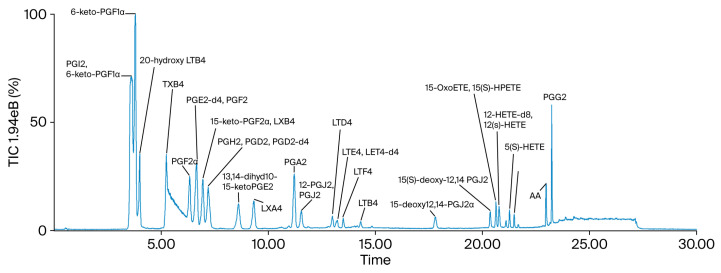
LC-MS/MS chromatogram of 28 eicosanoids performed on a triple quadrupole mass spectrometer with dynamic MRM in negative mode.

**Figure 6:**
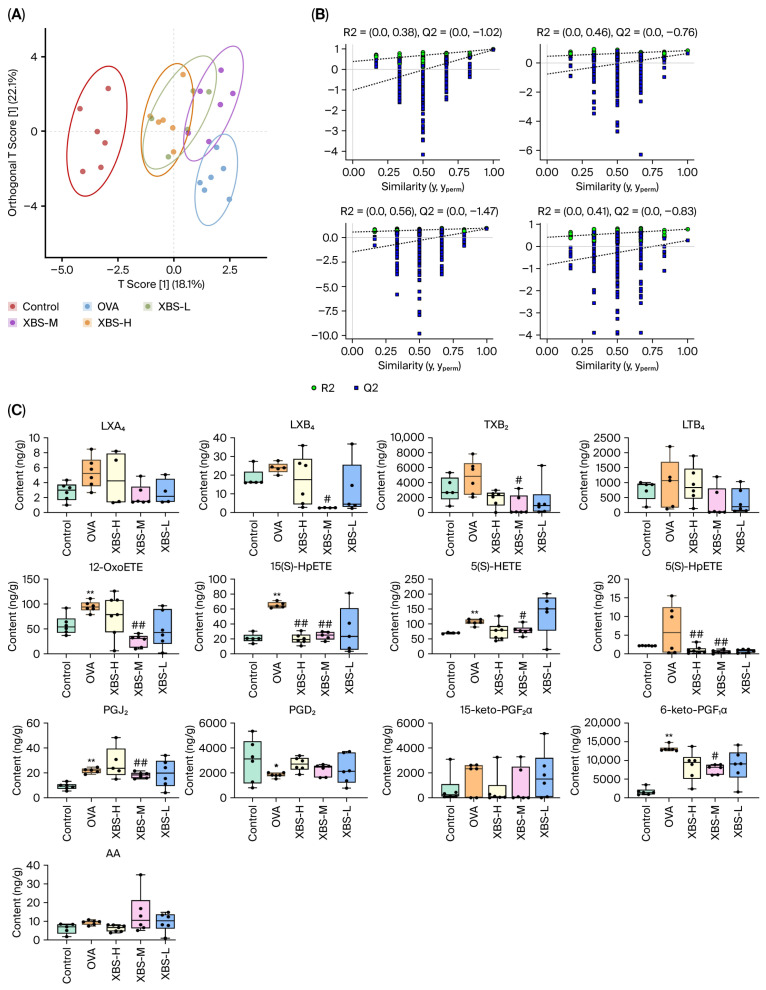
Determination and analysis of arachidonic acid and cascade metabolites (AAMs) in the lungs. (**A**) Partial least squares discriminant analysis (PLS-DA) score plot showing metabolic separation among control, OVA, and XBS treatment groups (XBS-L, XBS-M, XBS-H). (**B**) PLS-DA model validation. Left: score plot with 95% confidence ellipses; right: permutation test (200 iterations) confirming model robustness. (**C**) Effect of XBS on selected AAs in OVA-induced chronic allergic pulmonary inflammation mice (ng/g). mean ± SD (*n* = 6). * *p* < 0.05 and ** *p* < 0.01 vs. control; # *p* < 0.05 and ## *p* < 0.01 vs. OVA (one-way ANOVA, SNK test).

To investigate the effects of XBS on arachidonic acid metabolism, twenty-eight arachidonic acid metabolites (AAMs), including prostaglandins (PGs), leukotrienes (LTs), and hydroxyeicosatetraenoic acids (HETEs), were quantitatively measured in lung tissue samples collected 24 h after the final OVA challenge (day 19) using UPLC-MS/MS. Representative chromatograms of the AAMs are shown in Figure 5. Partial least squares discriminant analysis (PLS-DA) was performed to visualize metabolic differences among the control, OVA, and XBS-treated groups (Figure 6A,B). The PLS-DA results showed significant differences among the control group, OVA group, and XBS-treated groups (Figure 6A,B). The model quality parameters were accuracy = 0.95, R^2^X = 0.82, Q^2^ = 0.68, indicating reliable discrimination of metabolic differences.

Quantitative comparisons among groups at day 19 were performed using one-way ANOVA followed by appropriate post hoc tests. Compared with the control group, LTB_4_ levels increased by 15.1%, TXB_2_ by 51.6%, 15(S)-HpETE by 163.2%, and 12-OxoETE by 64.6%, indicating robust activation of leukotriene synthesis and oxidative lipid mediator production during chronic allergic inflammation. Notably, XBS administration markedly attenuated these alterations. Compared with the OVA group, XBS-M reduced LTB_4_ levels by 73.4%, TXB_2_ by 80.7%, 15(S)-HpETE by 63.6%, and 12-OxoETE by 71.7%. The pronounced suppression of leukotrienes and 12-HETE–related metabolites suggests that XBS preferentially targets LOX-mediated AA metabolism under chronic inflammatory conditions. (Figure 6C). These findings demonstrate that OVA induces allergic pulmonary inflammation via selective activation of leukotriene (LTB_4_, LTC_4_, LTD_4_) and 12-HETE pathways, whereas XBS exerts anti-inflammatory effects primarily by restoring these dysregulated metabolic pathways.

### 2.6. Modulation of Arachidonic Acid Metabolites by XBS in C48/80-Induced Allergic Pulmonary Inflammation

**Figure 7:**
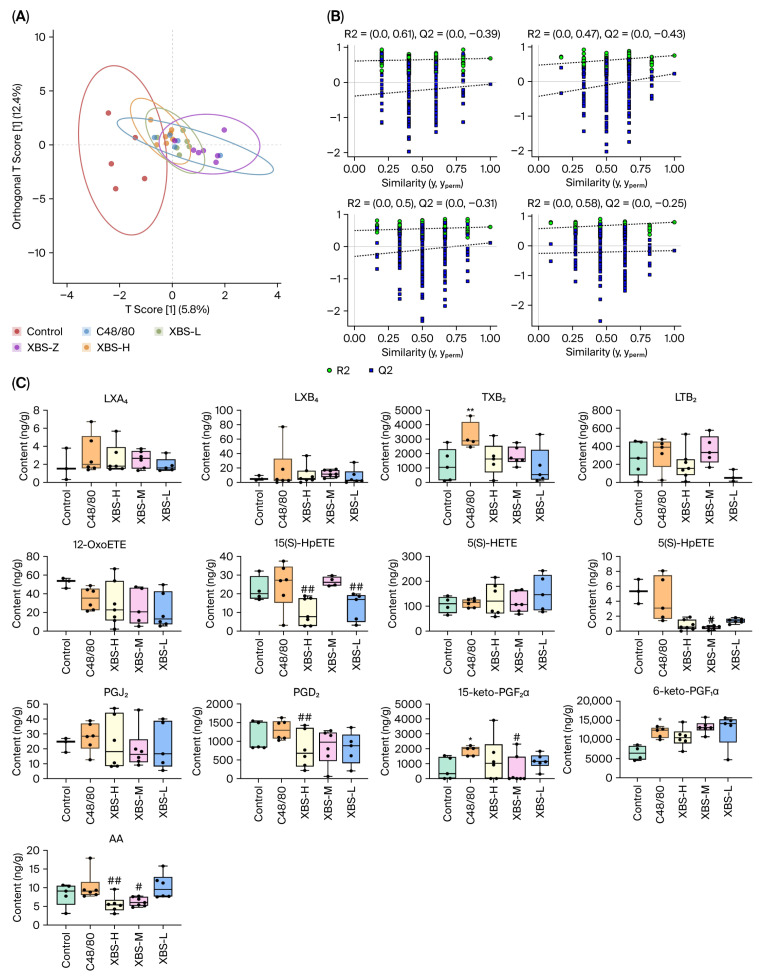
Determination and analysis of arachidonic acid and cascade metabolites (AAMs) in the lungs. (**A**) PLS-DA score plot showing metabolic separation among control, C48/80, and XBS treatment groups (XBS-L, XBS-M, XBS-H). (**B**) PLS-DA model validation. Left: score plot with 95% confidence ellipses; right: permutation test (200 iterations) confirming model robustness. (**C**) Effect of XBS on selected AAs in C48/80-induced acute allergic pulmonary inflammation mice (ng/g). mean ± SD (*n* = 6). * *p* < 0.05 and ** *p* < 0.01 vs. control; # *p* < 0.05 and ## *p* < 0.01 vs. OVA (one-way ANOVA, SNK test).

In the C48/80-induced allergic pulmonary inflammation model, lung tissue samples were collected 30 min after C48/80 injection following 7 days of XBS pretreatment for targeted metabolomic analysis. To assess global metabolic alterations, partial least squares discriminant analysis (PLS-DA) was performed among the control, C48/80, and XBS-treated groups (Figure 7A,B). The model quality parameters were accuracy = 0.95, R^2^X = 0.79, Q^2^ = 0.63, indicating reliable discrimination of metabolic differences. BS intervention predominantly modulated arachidonic acid metabolism, particularly affecting prostaglandin-related metabolites.

Quantitative analysis revealed predominant activation of the cyclooxygenase (COX) pathway following C48/80 stimulation. Compared with the control group, TXB_2_ levels increased by 119.4%, PGD_2_ by 17.7%, PGJ_2_ by 28.3%, and 15-keto-PGF_2_α by 183.8%. In contrast, free arachidonic acid (AA) levels increased more moderately, by 24.7%, suggesting rapid enzymatic conversion of AA into downstream prostaglandins during acute inflammatory responses. XBS treatment markedly attenuated these metabolic alterations. Compared with the C48/80 group, XBS-M reduced TXB_2_ levels by 29.7%, PGD_2_ by 34.6%, PGJ_2_ by 28.9%, and 15-keto-PGF_2_α by 70.1%. In addition, AA levels were decreased by 39.2%, indicating suppression of both substrate availability and downstream prostaglandin production. these findings demonstrate that C48/80-induced acute allergic pulmonary inflammation is characterized by rapid COX-dominant prostaglandin overproduction, whereas XBS effectively normalizes this metabolic burst through coordinated regulation of upstream AA release and downstream prostaglandin synthesis.

Overall, OVA challenge predominantly activated leukotriene and 12-HETE pathways, whereas C48/80 triggered overproduction of prostaglandins and 5-HETE. XBS effectively normalized these distinct metabolic disturbances, demonstrating differential modulation of arachidonic acid metabolism in chronic versus acute allergic inflammation.

### 2.7. Effect of XBS on the p38 MAPK Pathway in Mouse with Allergic Pulmonary Inflammation

**Figure 8:**
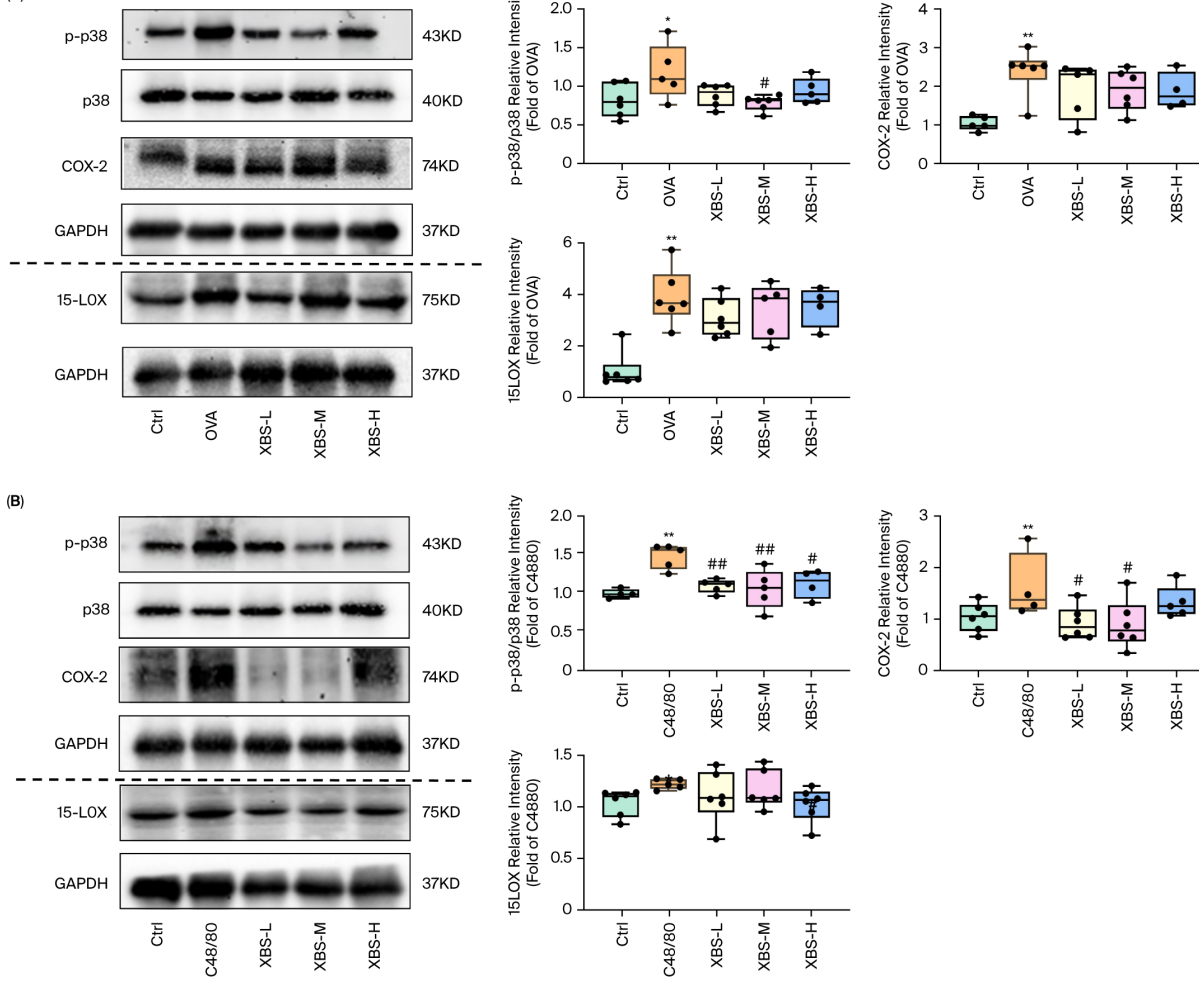
Effects of XBS on the p38 MAPK pathway in mice. (**A**) Representative Western blot images and quantitative analysis of p-p38 MAPK/p38 MAPK, COX-2, and 15-LOX protein expression in lung tissues collected 24 h after the final OVA challenge (day19). (**B**) Representative Western blot images and quantitative analysis of p-p38 MAPK/p38 MAPK, COX-2, and 15-LOX protein expression in lung tissues collected 30 min after C48/80 injection. Protein expression levels were normalized to GAPDH and presented as mean ± SD (*n* = 6 per group). Dashed lines indicate proteins detected on separate membranes due to the similar molecular weights of COX-2 (74 kDa) and 15-LOX (75 kDa). * *p* < 0.05 and ** *p* < 0.01 compared with the control group; # *p* < 0.05 and ## *p* < 0.01 compared with the model group.

To investigate the role of XBS in regulating the arachidonic acid metabolism (AAM) pathway in allergic pulmonary inflammation, we examined p38 MAPK phosphorylation status and the expression of downstream metabolic enzymes (COX-2 and 15-LOX) that directly catalyze AA conversion to prostaglandins and leukotrienes, respectively. In the OVA model, activation of the p38 MAPK pathway was evident, as demonstrated by a pronounced increase in the phospho-p38/p38 ratio (Figure 8A). This chronic, IgE-mediated response was accompanied by robust upregulation of the downstream lipoxygenase-related enzyme 15-LOX, consistent with the leukotriene- and HETE-dominant metabolic profile observed earlier. XBS administration significantly attenuated p38 MAPK activation and suppressed 15-LOX expression, indicating that XBS effectively inhibits LOX-associated inflammatory signaling in chronic allergic pneumonitis.

In contrast, the C48/80 model exhibited rapid activation of the p38 MAPK pathway together with marked induction of COX-2, reflecting the prostaglandin-driven acute inflammatory response characteristic of mast-cell degranulation. XBS treatment markedly reduced the phospho-p38/p38 ratio and downregulated COX-2 expression across groups (Figure 8B). These findings demonstrate that XBS attenuates acute inflammation primarily by inhibiting COX-2–associated signaling downstream of p38 MAPK.

## 3. Discussion

Allergic pulmonary inflammation is a highly prevalent and clinically refractory condition. Although current therapies provide symptomatic relief, they are limited by long-term dependence, adverse effects, and the emergence of therapeutic resistance, highlighting an urgent need for novel therapeutic strategies. This study provides comprehensive evidence that XBS exerts broad-spectrum anti-inflammatory effects across both acute and chronic allergic models, addressing a critical clinical need for treatments that can manage the heterogeneous nature of allergic lung diseases. Unlike conventional single-target therapies, XBS demonstrated multi-pathway regulation, encompassing suppression of IgE and histamine release, modulation of arachidonic acid metabolism, and inhibition of p38MAPK phosphorylation. These integrated effects highlight XBS as a promising candidate for allergic pulmonary inflammation, supporting its potential translational value for asthma and related disorders.

XBS is traditionally used to clear lung heat and relieve cough and wheezing, and is suitable for patients with mild heat-induced pulmonary symptoms [15]. Although the anti-inflammatory activities of individual components—such as flavonoids from *Cortex mori* and triterpenoid saponins from Glycyrrhizae Radix—are well documented [25,26], our data suggest that the whole formula produces differential modulation of arachidonic acid (AA) metabolic pathways that cannot be explained solely by additive effects of single constituents. Previous studies have reported multifaceted anti-inflammatory and immunomodulatory actions of XBS in models of pneumonitis, including suppression of pro-inflammatory mediators (e.g., IL-6, IL-8, TNF-α, ICAM-1) and reduction in eosinophil infiltration [27]. XBS has also been reported to ameliorate other allergic and inflammatory conditions such as allergic rhinitis and oxidative mucosal injury. However, its mechanism in allergic pulmonary inflammation remained unclear. Therefore, our study provides important mechanistic insights into how XBS ameliorates allergic pulmonary inflammation.

In the OVA-induced murine model of chronic allergic pulmonary inflammation, we observed key features reminiscent of human asthma, such as elevated IgE levels and inflammatory cell infiltration [28,29]. XBS markedly reduced allergen-specific IgE and inflammatory cell infiltration, consistent with previous observations in respiratory disease models [11,30]. The accompanying reduction in leukotriene (LTB_4_, LTC_4_, LTD_4_) and 12/15-HETE levels indicates suppression of LOX pathway overactivation, consistent with previous studies linking leukotrienes to airway hyperresponsiveness and fibrosis [31,32]. LTE_4_ has been implicated in sustaining airway inflammation and promoting persistent immune cell recruitment in chronic asthma models [33,34,35]. By reducing these metabolites, XBS may interrupt the self-perpetuating inflammatory amplification loop characteristic of chronic allergic responses. At the protein level, p38 MAPK is known to enhance cPLA_2_ phosphorylation, facilitating AA release and downstream eicosanoid biosynthesis [36,37,38]. Although cPLA_2_ was not directly measured in the present study, the observed reduction in p38 MAPK phosphorylation, together with decreased leukotriene and HETE levels (Figure 8), is consistent with attenuated upstream signaling in the AA metabolic cascade [39,40,41]. Previous studies have demonstrated that p38 MAPK inhibition reduces cPLA_2_-Ser505 phosphorylation and subsequent eicosanoid production in allergic inflammation models [42,43,44]. Recent studies have demonstrated that inhibition of p38 MAPK attenuates cPLA_2_ phosphorylation and downstream eicosanoid production in allergic inflammation models [45]. Based on these established mechanistic links and our metabolomic findings, we propose that XBS may indirectly modulate cPLA_2_ activity through suppression of p38 MAPK, although direct validation of cPLA_2_ phosphorylation remains to be confirmed.

In the C48/80-induced acute allergic pulmonary inflammation model, XBS exerted rapid anti-inflammatory effects via a distinct mechanistic pathway. C48/80 triggers immediate-type hypersensitivity via mast cell degranulation, leading to histamine and lipid mediator release [46]. XBS treatment significantly reduced histamine release and its downstream vascular effects, including vasodilation, increased capillary permeability, and smooth muscle contraction [47,48]. Metabolomic profiling revealed that XBS suppressed COX-derived prostaglandins (PGE_2_, PGD_2_, TXB_2_) in this model. These mediators collectively contribute to acute inflammatory responses: PGE_2_ promotes vasodilation and sensitizes nociceptors, PGD_2_ facilitates eosinophil recruitment, and TXB_2_ reflects thromboxane A_2_-mediated platelet activation and bronchoconstriction. It is important to note that PGE_2_ exerts context-dependent effects in inflammation. In the acute phase of C48/80-induced mast cell activation, PGE_2_ acts primarily as a pro-inflammatory mediator by promoting vasodilation and edema formation. Therefore, the XBS-mediated reduction in PGE_2_ in this model reflects attenuation of acute inflammatory responses rather than suppression of potential resolution-phase functions. This distinction reflects the temporal evolution of AA metabolism-prostaglandins dominate early vascular and contractile responses, while leukotrienes drive sustained tissue remodeling [49,50]. Therefore, XBS appears to exert a context-dependent regulatory effect rather than a uniform suppression of eicosanoid production. This context-dependent regulatory pattern suggests that XBS adapts its pharmacological action to the inflammatory milieu, such flexible modulation may represent a pharmacological advantage over single-target agents like selective COX or LOX inhibitors.

Collectively, the OVA and C48/80 models revealed fundamentally distinct inflammatory paradigms. The OVA model reflects a sustained adaptive immune–driven response characterized by IgE elevation and LOX-dominant leukotriene activation, whereas the C48/80 model represents a rapid mast cell–mediated inflammatory burst dominated by COX-derived prostaglandins and histamine release. These differences highlight temporal (chronic vs. acute) and biochemical (LOX vs. COX dominance) divergence between the two models. Notably, XBS demonstrated context-dependent modulation rather than uniform suppression of AA metabolism, preferentially attenuating leukotriene pathways in chronic inflammation while suppressing prostaglandin overproduction in acute settings.

While network pharmacology identified several potential signaling pathways, we prioritized experimental validation of the p38 MAPK-AA pathway based on its direct upstream position in AA metabolism. A key mechanistic observation from this study is the consistent suppression of p38 MAPK phosphorylation across both acute and chronic inflammation models, suggesting that this pathway may serve as an important upstream regulatory node. P38MAPK phosphorylates cPLA_2_, facilitating AA mobilization and eicosanoid biosynthesis [36,51,52,53]. Recent studies have further demonstrated that p38 MAPK activation directly regulates the transcriptional expression of COX-2 and 5-LOX through downstream transcription factors such as ATF-2 and CREB, thereby amplifying inflammatory lipid mediator production [54,55,56]. Consistent with previous reports, inhibition of the p38–cPLA2 axis reduces leukotriene and prostaglandin production in allergic airway inflammation [57,58], supporting p38MAPK as a viable anti-inflammatory target. Network pharmacology predicted MAPK pathway as a potential target pathway, which was partially supported by Western blotting results showing reduced p38MAPK phosphorylation. The observed changes in COX-2, 15-LOX and p38MAPK expression provide preliminary mechanistic insights into the anti-inflammatory effects of XBS while avoiding the pathway-specific limitations of conventional therapeutics. XBS alleviated allergic pulmonary inflammation, which was associated with reduced p38MAPK phosphorylation and downstream protein expression, suggesting involvement of this signaling pathway in its therapeutic effects.

Several limitations should be acknowledged. Firstly, murine models may not fully replicate human disease complexity, including genetic and environmental factors. Secondly, the dosing regimen was extrapolated from traditional use, requiring careful translation to human pharmacokinetics and safety standards. Thirdly, our study focused on short- to medium-term outcomes and did not evaluate long-term toxicity or pharmacodynamic stability. Fourthly, while our data support a potential role of p38 MAPK-mediated regulation of cPLA_2_ and downstream AA metabolism based on phosphorylation status and metabolite profiles, direct measurement of cPLA_2_ phosphorylation (Ser505) and enzymatic activity would provide more definitive mechanistic validation. Future studies should employ phospho-specific antibodies and enzymatic assays to directly confirm cPLA_2_ regulation by XBS. Lastly, while p38MAPK emerged as a key target, XBS likely modulates additional processes—such as epithelial barrier integrity, mucus secretion, or oxidative stress responses—that warrant future investigation. Clinical trials and systems pharmacology are needed to determine optimal dosing, confirm safety, and explore broader mechanisms.

## 4. Materials and Methods

### 4.1. Materials and Reagents

OVA (Batch No. 326A0522) was purchased from Beijing Solarbio Science & Technology Co., Ltd. (Beijing, China). Aluminum hydroxide adjuvant (Lot No. 202411057) was obtained from Huking Industry (Shanghai, China). Dexamethasone (H33020822) were obtained from Anhui Golden Sun Biochemical Pharmaceutical Co., Ltd. (Anhui, China). Enzyme-linked immunosorbent assay (ELISA) kits for interleukin-4 (IL-4, Lot No. V01026009), interleukin-6 (IL-6, Lot No. V12026404), interleukin-1β (IL-1β, Lot No. U28026479), and tumor necrosis factor-α (TNF-α, Lot No. V02026010) were obtained from Wuhan Huamei Bioengineering Co. (Wuhan, China). OVA-sIgE (Lot No. m1063583) and histamine (HIS, JL45802) assay kits were obtained from Shanghai Enzyme-linked Biotechnology Co. (Shanghai, China). Methanol (MS grade), formic acid (MS grade), acetonitrile (MS grade) and isopropanol (MS grade) were purchased from Fisher Scientific (Fair Lawn, NJ, USA). Antibodies against p38MAPK (8690), Phospho-p38MAPK (Thr180/Tyr182) (4511), COX-2 (12282), and rabbit polyclonal antibody against glyceraldehyde-3-phosphate dehydrogenase (GAPDH), were obtained from Cell Signaling Technology (Danvers, MA, USA). Antibody against 15-LOX (Cat No. 13286-1-AP) was obtained from Proteintech Group, Inc. (Rosemont, IL, USA). *Cortex mori*, *Lycii Radicis Cortex*, and *Glycyrrhizae Radix et Rhizoma* were purchased from Tong Ren Tang (Group) Co., Ltd. in Beijing, China in April 2025 and authenticated as genuine.

### 4.2. Extraction of Experimental Samples

In accordance with the “Ancient Classic Formulas Key Information Table (25 formulas)”, *Lycii Radicis Cortex* (90 g), roasted *Cortex mori* (90 g), *Glycyrrhizae Radix et Rhizoma* (9 g), and japonica rice (63 g) were weighed and placed in a decoction vessel. After adding 900 mL of water, the mixture was boiled uncovered for 8 min, then covered and decocted for 54 min. The decoction was filtered through a No. 9 sieve and concentrated by rotary evaporation.

### 4.3. Identification of Major Chemical Constituents in XBS

An aliquot of 0.5 mL of each sample solution was transferred into a 2 mL centrifuge tube. After adding 1 mL of methanol, the mixture was vortexed for 10 min to ensure homogeneity. The sample was then centrifuged at 12,000 rpm (relative centrifugal force ~1370× *g*, with a radius of 3.8 cm) for 10 min at 4 °C. The resulting supernatant was collected and filtered through a 0.22 μm membrane for subsequent LC-MS analysis.

#### 4.3.1. Chromatographic Condition

Chromatographic separation was performed on a Waters BEH C18 (50 mm × 2.1 mm, 1.7 μm) maintained at 30 °C. The mobile phase consisted of (A) 0.1% formic acid in water and (B) acetonitrile, delivered at a flow rate of 0.3 mL/min. The gradient elution program was set as follows: 5% to 30% B (0–6 min), 30% to 78% B (6–12 min), 78% to 95% B (12–17 min), and 95% to 5% B (17–20 min). The injection volume was 2 μL.

#### 4.3.2. Mass Spectrometry

Analysis was conducted using an electrospray ionization (ESI) source operating in both positive and negative ionization modes. The key MS parameters were set as follows: heater temperature, 325 °C; sheath gas flow, 45 arb; auxiliary gas flow, 15 arb; sweep gas flow, 1 arb; spray voltage, 3.5 kV; capillary temperature, 330 °C. Full-scan MS data were acquired over a mass range of *m*/*z* 150–1000.

### 4.4. Animal Experiments

Seventy-two specific pathogen-free (SPF) male ICR mice (6–8 weeks old, 20 ± 2 g) were obtained from Beijing Vital River Laboratory Animal Technology Co., Ltd. (Certificate No. SYXK (Beijing, China) 2012-0006). Animals were housed in the SPF-grade animal facility of the Institute of Chinese Materia Medica, China Academy of Chinese Medical Sciences. Environmental conditions were maintained at 23 ± 2 °C, 50 ± 10% relative humidity, under a 12 h light/12 h dark cycle. The experimental protocol was approved by the Animal Care and Use Ethics Committee of the Institute of Chinese Materia Medica, China Academy of Chinese Medical Sciences (Ethical Approval No. 2024B390). Mice had free access to purified water and a standard rodent pellet diet.

The 72 mice were randomly allocated to two separate experimental series: the OVA-induced chronic inflammation model and the C48/80-induced acute inflammation model, each comprising 36 mice (*n* = 6 per group).

For the OVA model, mice were randomly assigned to six groups (*n* = 6/group): control, OVA (5 mg/kg), OVA + DXMS (10 mg/kg), and OVA + XBS-L (1.25 g/kg), OVA + XBS-M (2.5 g/kg), and OVA + XBS-H (5 g/kg). XBS doses represented 0.5×, 1×, and 2× the clinical equivalent dose, respectively. On days 1, 3, and 5, all groups except the control group were sensitized by intraperitoneal injection of OVA mixed with aluminum hydroxide adjuvant, whereas the control group received saline. From days 14 to 18, the control group was intranasally instilled with PBS, while the remaining groups were challenged with OVA (50 μL, 10 mg/kg) to induce airway inflammation. Starting on day 6 and continuing for 14 consecutive days until day 21, the animals from OVA + XBS and OVA + DXMS groups received different doses of XBS or DXMS daily by oral gavage (10mL/kg body weight). The mice from control and OVA model groups were given an equal volume of saline. All animals were euthanized 24 h after the final OVA challenge, and plasma and lung tissues were collected for analysis.

For the C48/80 model, mice were assigned to six groups (*n* = 6/group): control, C48/80 (5 mg/kg), C48/80 + DXMS (10 mg/kg), C48/80 + XBS-L (1.25 g/kg), C48/80 + XBS-M (2.5 g/kg), and C48/80 + XBS-H (5 g/kg). Seven days prior to modeling, mice in the C48/80 + XBS and C48/80 + DXMS groups received daily oral administration of the corresponding agents (10 mL/kg body weight), while the control group received saline. On the day of modeling, the mice from the control group were injected with saline via the tail vein, whereas animals from all other groups received C48/80 injection via tail vein (10 mL/kg body weight). Animals were observed for 30 min after C48/80 stimulation and then euthanized. Lung tissues were collected for histopathological evaluation, cytokine quantification, and subsequent assays.

All animals were euthanized humanely using carbon dioxide (CO_2_) inhalation, applied at a controlled flow rate corresponding to 30–50% of the chamber volume per minute, followed by cervical dislocation to ensure complete death. All procedures were performed in strict accordance with the AVMA Guidelines for the Euthanasia of Animals.

### 4.5. Determination of White Blood Cell Counts and Plasma IgE/Histamine Levels

Whole blood was collected from mice in each group and anticoagulated with ethylenediaminetetraacetic acid (EDTA). Total white blood cell (WBC) counts and WBC differential counts (including NEUT, LYMPH, MONO, EO, BASO) were determined using a fully automated hematology analyzer. After centrifugation, plasma was separated for subsequent measurement of IgE and histamine (HIS) levels using ELISA.

### 4.6. ELISA Determination of Inflammatory Factors in Lung Tissue

Lung tissues from mice were homogenized on ice with pre-chilled phosphate-buffered saline (PBS) at a weight-to-volume ratio of 1:10 (g/mL) using a tissue homogenizer. The homogenates were subjected to repeated freeze–thaw cycles. Following centrifugation, the resulting supernatant was collected, and the contents of IL-1β, IL-4, IL-6 and TNF-α were detected in the supernatant of lung tissues according to the instructions of the ELISA kit.

### 4.7. Histopathological Examination

All samples were fixed with neutral-buffered formalin and embedded in paraffin. Sections (4 μm thick) were prepared and stained with hematoxylin and eosin (HE) for histopathological evaluation. To evaluate pulmonary pathology, five fields per tissue section were scored using semi-quantitative grading methods as follows. The degree of lung injury was scored according to established criteria [59]. Tissue injury was graded on a scale of 1–5 for: (1) alveolar congestion, (2) hemorrhage, (3) neutrophil infiltration in alveolar or vessel walls, and (4) alveolar wall thickness. The 1–5 scale corresponded to: 1 = slight injury, 2 = mild injury, 3 = moderate injury, 4 = severe injury, and 5 = most severe injury (see Table 2 for scoring criteria).

### 4.8. Network Pharmacology

To explore and predict the potential targets of XBS in allergic pulmonary inflammation, a network pharmacology approach was applied. The keywords “Sang Bai Pi”, “Di Gu Pi”, and “Zhi Gan Cao” were searched in the Traditional Chinese Medicine Systems Pharmacology Database (TCMSP). Their Canonical SMILES were obtained from PubChem (https://pubchem.ncbi.nlm.nih.gov/ accessed on 1 July 2025) and used for target prediction (probability > 0) in Swiss Target Prediction (http://www.swisstargetprediction.ch/ accessed on 1 July 2025), with the species attribute set to “Homo sapiens”. Potential targets related to pneumonia and allergic were retrieved from OMIM (https://omim.org/ accessed on 10 July 2025) and GeneCards (https://www.genecards.org/ accessed on 20 July 2025). Venny analysis was used to identify the intersection between the potential targets of XBS and those of pneumonia and allergies. These overlapping targets were considered potential therapeutic targets of XBS for pneumonia and allergies. The protein–protein interaction (PPI) network of the intersected targets was constructed using the STRING 11.0 database (https://string-db.org, accessed on 30 July 2025). Targets in the network with a degree greater than 3 times the median were defined as core targets. Subsequently, the “drug-active ingredient-target” network of XBS was visualized using Cytoscape 3.7.2 software. Gene Ontology (GO) functional annotation and Kyoto Encyclopedia of Genes and Genomes (KEGG) pathway enrichment analyses for the intersected targets were performed using the DAVID 6.8 database.

### 4.9. Determination of AAMs in Lungs by UPLC-MS/MS

Approximately 30 mg of lung tissue was homogenized in 600 μL of methanol: acetonitrile (5:3, *v*/*v*) containing 0.01 M butylated hydroxytoluene and 6 μL of formic acid. The homogenates were centrifuged at 15,000× *g* for 15 min at 4 °C, and the resulting supernatants were dried under a nitrogen stream. The residues were reconstituted in 100 μL of methanol containing internal standards (PGE_2_-d_4_, LTE_4_-d_5_, and 12-HETE-d_8_). Chromatographic separation was performed on an ACQUITY UPLC BEH C18 column (100 mm × 2.1 mm, 1.7 μm, Waters) maintained at 40 °C. The mobile phases consisted of A (0.1% formic acid in water) and B (acetonitrile/isopropanol, 90:10, *v*/*v*), with a flow rate of 0.45 mL/min. The 30 min gradient program was as follows: 0–1 min (25% B), 1–4 min (25–33% B), 8–14.5 min (33–48% B), 19–23 min (52–95% B), and 23.5–30 min (25% B). Targeted detection of AAMs was performed on a UPLC-TQS-MS/MS system in MRM mode under negative ESI conditions (capillary voltage: 2.5 kV; source temperature: 150 °C; desolvation temperature: 600 °C; desolvation gas flow: 1000 L/h; cone gas flow: 150 L/h). Quantification was achieved using internal-standard calibration, and data processing was performed using TraceFinder 4.1 software.

### 4.10. Western Blotting Analysis

Lung tissues were collected at the 24 h after the final OVA challenge or 30 min after C48/80 injection and homogenized in RIPA lysis buffer containing protease and phosphatase inhibitors. After centrifugation, the supernatants were collected as total protein extracts, quantified, and equal amounts of protein were subjected to SDS-PAGE. Proteins were separated by sodium dodecyl sulfate-polyacrylamide gel electrophoresis (SDS-PAGE) on 8–12% gels and electrotransferred onto polyvinylidene fluoride (PVDF) membranes. Membranes were blocked with 5% skimmed milk in Tris-buffered saline containing 0.1% Tween-20 (TBST) for 2 h at room temperature. Subsequently, membranes were incubated overnight at 4 °C with the following primary antibodies: p38MAPK (1:500), Phospho-p38MAPK (1:500), COX-2 (1:500), 15-LOX (1:500). After washing with TBST, membranes were incubated with HRP-conjugated goat anti-rabbit IgG secondary antibody for 2 h at room temperature. Protein bands were visualized using chemiluminescence detection reagent, and quantified using Image-Pro Plus 6.0 software. GAPDH served as the loading control for normalization.

### 4.11. Statistical Analysis

Quantitative data were statistically processed using Graphpad Prism 9.0 software, and the data of each group were expressed as mean ± SD. Normality was assessed using the Shapiro–Wilk test, and homogeneity of variance was evaluated via Levene’s test. For multiple group comparisons, one-way analysis of variance (ANOVA) was employed, followed by Student-Newman-Keuls (SNK) post hoc test to identify specific between-group differences. All experiments were independently replicated at least three times to ensure reproducibility.

## 5. Conclusions

In summary, this study demonstrates that XBS alleviates both acute and chronic allergic pulmonary inflammation by suppressing IgE and histamine release, reducing inflammatory cytokines, and modulating arachidonic acid metabolism via inhibition of p38 MAPK. These findings highlight the therapeutic potential of XBS as a multi-target intervention for allergic lung diseases. Further studies addressing clinical dosage, long-term safety, and additional mechanisms are necessary to support its translation into clinical practice.

## Figures and Tables

**Table 1:** Identification results of Chemical compounds of XBS.

Ret. Time	Formula	Compound	(+) ESI-MS			(−) ESI-MS			Type	Herbal Source
			Measured Value	Theoretical Value	PPM	Measured Value	Theoretical Value	PPM		
9.25	C_42_H_62_O_16_	Glycyrrhizic acid *	823.4130	823.4111 [M+H]^+^	2.31	821.3954	821.3965 [M−H]^−^	−1.33	Triterpenoid saponins	*Glycyrrhizae Radix et Rhizoma*
4.90	C_21_H_22_O_9_	Liquiritin (SH) *	419.1352	419.1337 [M+H]^+^	3.57	417.1208	417.1191 [M−H]^−^	4.07	Flavonoid	*Glycyrrhizae Radix et Rhizoma*
3.07	C_16_H_18_O_9_	Chlorogenic acid *	355.1051	355.1024 [M+H]^+^	7.60	353.0880	353.0878 [M−H]^−^	0.56	Organic acids	*Cortex mori/Lycii Radicis Cortex*
2.58	C_28_H_42_N4O_6_	KukoaMine B *	531.3188	531.3177 [M+H]^+^	2.07	529.3024	529.3032 [M−H]^−^	−1.51	Alkaloid	*Lycii Radicis Cortex*
2.78	C_26_H_32_O_14_	Mulberroside A *	569.1888	569.1865 [M+H]^+^	4.04	567.1707	567.1719 [M−H]^−^	−2.11	Others	*Cortex mori*

* Compounds identified by comparison with authentic standards.

**Table 2:** Semi-quantitative grading system for lung tissue injury assessment.

Pathological Grade	Special Description of Pathological Changes	Score
−	No significant alveolar wall abnormality, no inflammatory infiltration	1
+	Focal thickening of individual alveolar walls with occasional scattered infiltration of a few inflammatory cells	2
++	Focal thickening of small areas of alveolar wall with occasional scattered infiltration of a few inflammatory cells	3
+++	Large thickening of the alveolar wall, large inflammatory cell infiltration, and loss of some alveolar lumen structures	4
++++	Diffuse thickening of the alveolar wall, infiltration of inflammatory cells, basic loss of alveolar luminal structure, occasional necrotic shedding of alveolar wall epithelial cells	5

“−“, ”+”, “++”, “+++”, and “++++” indicate increasing severity of lung tissue injury corresponding to scores of 1, 2, 3, 4, and 5, respectively.

## Data Availability

The original contributions presented in this study are included in the article. Further inquiries can be directed to the corresponding author.

## Abbreviations

The following abbreviations are used in this manuscript:

AA	Arachidonic acid
AAMs	Arachidonic acid metabolites
BASO	Basophil
C48/80	Compound 48/80
COX	Cyclooxygenases
DXMS	Dexamethasone
EDTA	Ethylenediaminetetraacetic acid
EO	Eosinophil
ELISA	Enzyme-linked immunosorbent assays
HIS	Histamine
HETEs	Hydroxy eicosatetraenoic acids
IgE	Immunoglobulin E
LOX	Lipoxygenase
LYMPH	Lymphocyte
MONO	Monocyte
NEUT	Neutrophil
OPLS-DA	Orthogonal Partial Least Squares-Discriminant Analysis
OVA	Ovalbumin
UPLC-MS/MS	UltraPerformance Liquid Chromatography–Tandem Mass Spectrometry
WBC	White Blood Cell
XBS	Xiebai san
